# Subthreshold Micropulse Laser (577 nm) Therapy with an Individual Approach for Power Titration in Patients with Chronic Central Serous Chorioretinopathy (Pilot Study)

**DOI:** 10.1155/2024/9750395

**Published:** 2024-10-08

**Authors:** Taras Kustryn, Oleg Zadorozhnyy, Illia Nasinnyk, Nataliya Pasyechnikova, Andrii Korol

**Affiliations:** State Institution The Filatov Institute of Eye Diseases and Tissue Therapy of the National Academy of Medical Sciences of Ukraine, Frantsuzskiy Bulvar, 49/51, Odesa 65061, Ukraine

## Abstract

**Purpose:**

To study the safety and effectiveness of subthreshold micropulse (SML) 577 nm laser therapy with an individual power titration in treating patients with chronic central serous chorioretinopathy (CSC).

**Methods:**

The study was a prospective, single-centre observation of 30 patients (30 eyes) with chronic CSC. All patients with chronic CSC were treated with a 577 nm micropulse laser. Individual power parameters for each patient were titrated in a micropulse mode. The primary outcome measure was best-corrected visual acuity (BCVA) at 6-month follow-up. The secondary outcome measures were as follows: central retinal thickness (CRT) and maximum subretinal fluid height (SRFH) according to optical coherence tomography, number of laser sessions, and treatment safety at 6-month follow-up.

**Results:**

Before treatment, the mean BCVA was 0.35 ± 0.16, the mean CRT was 285 ± 76 µm, and the mean SRFH was 311 ± 85 µm. Six months after 577 nm SML therapy, there was a statistically significant increase in the mean BCVA with the maximum correction to 0.45 ± 0.15 (*p* = 0.001). The mean CRT and SRFH decreased significantly to 236 ± 45 *μ*m (*p* = 0.003) and 45 ± 25 *μ*m (*p* = 0.001), respectively. At the end of the follow-up, complete resorption of the subretinal fluid was noted in 50% (15 eyes), and in 43% (13 eyes), a decrease in the SRFH was observed. During the entire observation period, 25 patients underwent 1 session of 577 nm SML therapy, 2 patients underwent 2 sessions, and 3 patients underwent the intervention three times.

**Conclusion:**

SML 577 nm therapy with individual selection of laser power in a micropulse mode is a safe and effective method of treating patients with chronic CSC. Further studies are needed to test the long-term safety and efficacy of 577 nm SML therapy with individual power settings in chronic CSC.

## 1. Introduction

Central serous chorioretinopathy (CSC) is a common chorioretinal disease, clinically manifested by secondary serous detachment of the neurosensory retina due to dysfunction of the retinal pigment epithelium (RPE) and accumulation of subretinal fluid (SRF). CSC has an unclear aetiology and often recurrent course. The disease may be asymptomatic in the early stages with minor structural changes in the choroid and retina. With a long-term recurrent course of CSC, fundus morphological changes appear in the form of degeneration of the RPE and neurosensory retina [1]. Currently, there is no widely accepted classification of CSC. The most common division of CSC into acute and chronic forms is based on the duration of persistence of subretinal fluid and the presence of changes in the RPE [2]. This disease has a variety of clinical manifestations and lacks a uniform treatment approach due to an incomplete understanding of its pathogenesis [3].

Photodynamic therapy (PDT) with verteporfin is a pathogenetically substantiated method of treating chronic CSC aimed at the choroid [4]. PDT is effective due to temporary choriocapillaris hypoperfusion and long-term choroidal remodelling, which leads to a decrease in choroidal overload and vascular hyperpermeability, especially in the case of RPE decompensation [5–7]. However, PDT in chronic CSC can result in the development of a variety of complications: choroidal ischemia, choroidal atrophy, RPE detachment, subretinal neovascularization, as well as atrophy of the RPE and photoreceptors [8–10]. All these complications may cause a rapid and irreversible decline in vision and limit PDT use in patients with high visual function. In addition, the global shortage of verteporfin since July 2021 has further limited the use of PDT in patients with CSC [11].

Subthreshold micropulse laser (SML) therapy is also widely used to treat patients with chronic CSC [12]. Unlike PDT, the mechanism of SML therapy is based on a sublethal effect on RPE cells to increase their functional activity. It is believed that one of the main mechanisms of action of SML is the thermal stimulation of the RPE to produce “heat shock proteins,” which leads to changes in its metabolism and improvement of its function [13]. However, there is no unified approach to subthreshold laser treatment in the micropulse mode and the issue of dosing laser energy when using this technique remains unresolved [14, 15].

Therefore, the purpose was to study the effectiveness and safety of subthreshold micropulse 577 nm laser therapy with individual power titration in treating patients with chronic central serous chorioretinopathy.

## 2. Methods

This prospective, single-centre pilot study included 30 patients (30 eyes) with chronic CSC and was approved by the Bioethical Committee of the State Institution (The Filatov Institute of Eye Diseases and Tissue Therapy of the National Academy of Medical Sciences of Ukraine) following the Declaration of Helsinki. All participants signed informed consent.

Inclusion criteria were as follows: active chronic CSC, which is defined as serous neurosensory retinal detachment on optical coherence tomography (OCT) with focal or diffuse areas of leakage on retinal fluorescein angiography (FA). Before inclusion in the study, all patients with chronic CSC were monitored for 3 months or longer to ensure spontaneous disease resolution.

Exclusion criteria were as follows: prior laser treatment for CSC, glaucoma or suspected glaucoma, intraocular or periocular inflammation, other diseases of the macula that may affect visual acuity (e.g., age-related macular degeneration, retinal vascular occlusion, diabetic retinopathy, epiretinal membrane, and any hereditary disease of the retina), and high myopia of ≥−6.00 diopter spherical equivalent. Extraocular exclusion criteria included the following: the use of systemic corticosteroids, pregnancy, antibiotic use, alcohol use, untreated hypertension, systemic diseases that could affect the eye, and inability to obtain patient consent.

All participants received detailed information regarding their disease, the study, and treatment. They were then allowed to consider participating in the study and consent was obtained.

Each patient underwent a basic examination, which included the following: best-corrected visual acuity (BCVA) testing, intraocular pressure measurement, biomicroscopy, dilated fundus examination, OCT (SOCT Copernicus OPTOPOL Technology, S.A., Zawiercie, Poland), and FA (TRC 50-EX, Topcon, Tokyo, Japan).

All patients with active chronic CSC were treated with a 577 nm laser (Supra, Quantel Medical, Sedan, France). Ocular Mainster (standard) focal/grid contact lens (laser spot magnification ×1.05; Ocular Instruments, Bellevue, Washington, USA) was used. All patients received laser treatment in monospot mode with the following parameters: spot size of 100 *μ*m, exposure time of 0.2 s, and duty cycle of 9%. Individual power parameters for each patient were titrated on the normal retina in the region of the vascular arcades in the micropulse mode. Power titration was started at 500 mW and then gradually increased until a barely noticeable burn appeared, after which the power was reduced by 50% [16, 17]. Well-defined leakage points in midphase FA images (including the subfoveal location) were treated with a targeted focal laser without overlapping the intact retina in monospot mode. In the case of diffuse leakage areas, confluent laser therapy was applied over the entire lesion area without overlapping intact RPE in monospot mode following FA.

In all cases, patients underwent general ophthalmological, OCT and FA examinations before treatment. After that, there were monthly checkups for six months. Comprehensive examinations included BCVA, intraocular pressure measurement, dilated pupillary examination, and OCT, which were repeated at each follow-up visit. FA was repeated in cases of increased SRF based on OCT data and/or decreased visual acuity. The SML therapy was administered again as the maximum SRF height (SRFH) increased to more than 30 *μ*m or/and in the presence of actively leaking areas on repeated FA images.

The primary outcome was BCVA at 6 months of follow-up. Secondary outcomes were as follows: central retinal thickness (CRT), maximum SRFH according to OCT, number of SML sessions, and treatment safety at 6 months of follow-up. The CRT and SRFH were manually measured with a caliper tool during each OCT visit. The CRT was measured from the vitreoretinal interface to the top level of the subretinal fluid perpendicularly in the centre of the fovea. The SRFH was measured from the top level of the subretinal fluid to RPE perpendicularly in the centre of the fovea. The SRFH was measured perpendicularly from the top level of the subretinal fluid to the RPE at the site of the maximum subretinal fluid level in the macula.

Statistical analysis was performed using Statistica 10.0 for Windows (StatSoft, Tulsa, OK, USA). In the current study, the mean ± standard deviation was used to describe patient characteristics. Analysis of variance (ANOVA) was performed to study changes in BCVA, CRT, and SRFH after treatment during the entire observation period. The significance level was set at *p* < 0.05.

## 3. Results

Thirty patients (30 eyes) completed the 6-month follow-up. The mean age of the patients was 43 ± 14 years, and 90% (27 patients) were male. All patients had areas of leakage in the macular region, either focal or diffuse, as observed in the FA. Before treatment, the mean BCVA was 0.35 ± 0.16, the mean CRT was 285 ± 76 *µ*m, and the mean SRFH was 311 ± 85 *µ*m.

**Figure 1:**
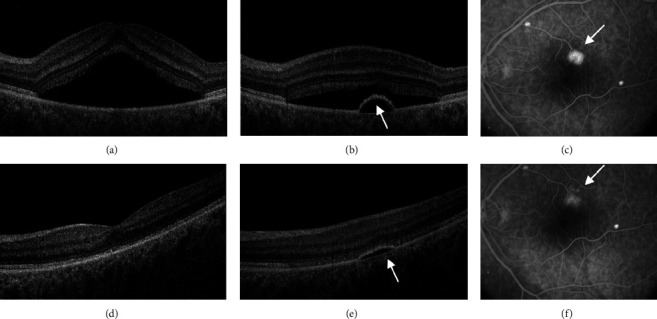
Multimodal imaging of a 45-year-old man with chronic central serous chorioretinopathy (CSC) who had one session of subthreshold micropulse laser (SML) therapy. Before treatment, (a) spectral domain optical coherence tomography (SD-OCT) shows the serous detachment of the neurosensory retina, (b) SD-OCT of the same patient shows juxtafoveal local retinal pigment epithelial detachment (PED) (white arrow), and (c) fluorescein angiography (FA) in the late phase demonstrates staining in the area of PED before treatment. One month after session of SML therapy, (d) SD-OCT shows improvement in SRF in fovea, (e) SD-OCT shows reduced retinal PED after treatment, and (f) FA in the late phase demonstrates regression of PED (white arrow).

After one month of SML therapy, complete resorption of the SRF occurred in 33% of patients (10 eyes) (Figure 1), while in the other 33% (10 eyes), the SRFH decreased. In 17% of cases (5 eyes), the maximum height of SRF remained unchanged, while in another 17% (5 eyes), it increased. In the general group, there was a significant decrease in the mean SRF after 1 month of follow-up from 311 ± 85 *μ*m to 185 ± 72 *μ*m (*p*=0.03). The mean CRT also decreased significantly from 285 ± 76 *μ*m to 264 ± 53 *μ*m (*p*=0.01). After the first SML session, the mean BCVA did not change and was 0.3 ± 0.1 (*p*=0.07).

In the third month of follow-up, 43% of patients (13 eyes) exhibited complete resorption of the SRF, and 11 eyes (37%) showed a decrease in SRFH. Repeated SML treatment was carried out in all cases where an increase in SRFH was noted (20%, 6 eyes). At this stage of follow-up, the mean CRT and the mean SRFH decreased statistically significantly to 246 ± 47 *μ*m and 80 ± 45 *μ*m, respectively (*p* < 0.05). The mean BCVA increased significantly to 0.4 ± 0.14 (*p*=0.03).

A significant increase in mean BCVA was noted 6 months after the study began, with values rising from 0.35 ± 0.16 to 0.45 ± 0.15 (*p*=0.001). At the 6-month follow-up, the mean CRT and mean SRFH significantly decreased to 236 ± 45 *μ*m (*p*=0.003) and 45 ± 25 *μ*m (*p*=0.001), respectively (Table 1).

At the end of the observation, complete resorption of the SRF was noted in 50% (15 eyes), and in 43% (13 eyes), a decrease in SRFH was observed. In 2 eyes (7%), the SRFH increased from initial values. Two patients (2 eyes) experienced CSC recurrence during the observation period.

In the presented study, 24 patients underwent one SML session, 3 patients underwent 2 sessions, and 3 patients underwent the SML therapy three times.

According to ophthalmoscopy, OCT, and FA, no case of damage to the retina was detected after SML therapy. In 5 patients, local RPE scars were identified at the site of titration of the threshold laser power.

## 4. Discussion

The issue of dosing laser power that is safe and sufficient for the therapeutic effect of subthreshold laser on the RPE in the absence of visible fundus changes remains unresolved. Analysis of previous studies of subthreshold 577 nm laser therapy revealed different points of view on this technique [14, 15, 17–26].

Currently, there is no single approach to laser exposure in the micropulse mode for SML therapy in patients with chronic CSC. The theory of enhanced photostimulation of the retina involves a low-intensity, high-density micropulse laser with fixed power settings, ensuring activation of “heat shock proteins” in the RPE. Gawęcki and colleagues successfully used this SML technique, densely applying 577 nm laser applications with a fixed power (250 mW) and a 5% duty cycle to the entire area of serous retinal detachment in patients with chronic CSC [22]. The authors reported complete SRF resorption in 70.6% of cases and a slight improvement in BCVA of approximately one line on the Snellen chart.

Other authors follow the laser power titration approach for SML therapy in patients with chronic CSC. Yadav and colleagues for the treatment of CSC carried out power selection for each patient individually by achieving a barely visible burn in the continuous mode of micropulse yellow laser (this power corresponded to 100%). However, during SML treatment, power values decreased by 50% [25]. Another power titration protocol for SML treatment was used by Inagaki and Maruko. The authors proposed to select the power when performing test photocoagulation outside the vascular arcades before the appearance of noticeable burns in the continuous mode of the laser. The procedure was performed in the micropulse mode using a 15% duty cycle at 200% threshold power with a pulse duration of 0.2 seconds. Such parameters of the laser allowed the release of 60% of the threshold power [20, 26]. This approach appears controversial, since in this case, not only the laser power is titrated but also the laser exposure changes, which makes the effect of the laser interaction with the retina less predictable.

We believe that the individual titration of laser power in the micropulse mode for SML therapy is the most rational. This technique of power titration consists of searching for a threshold value of power immediately in the micropulse mode, followed by its reduction during the therapeutic mode. In this case, laser-RPE interaction in gentle SML therapy in the absence of visible fundus burns becomes more predictable. This technique makes it possible to regulate thermal damage to RPE cells by changing the percentage of laser power from threshold values. This approach was mathematically substantiated and tested in an experiment to study the retinal ultrastructure after the interaction of 577 nm laser radiation with the RPE [16, 17].

In the study of Scholz and colleagues, power titration was carried out individually for each patient with chronic CSC in the intact retina next to the leakage zone in the micropulse mode. Thus, the selection of energy continued until the appearance of a visible burn, and after reaching the threshold, the laser power was reduced by 50% [14]. The authors used standardized treatment parameters: the multispot mode (without spacing between the spots), the spot size of 160 *μ*m, the exposure time of 0.2 s, and a duty cycle of 5%. Confluent SML therapy in the multispot mode was used for treating the hyperfluorescent areas on midphase indocyanine green angiography (ICGA) and the hot spots on midphase FA. The authors reported that 577 nm SML therapy was associated with the resolution or significant reduction of SRF in 75% of chronic CSC patients.

Chhablani and coauthors, in their subthreshold laser therapy guidelines for retinal diseases, suggest subthreshold laser with individual power titration as the first line of CSC patients' treatment. Their protocol recommends the following settings for subthreshold laser application: 5% duty cycle, 200 ms pulse duration, and 100–200 *μ*m spot size. Settings are the same for both acute and chronic CSC with no spacing between the spots using an integrated pattern system. The authors suggest titration with 50% of threshold power (threshold estimation using the subthreshold laser mode). For chronic CSC, the authors recommend confluent treating areas of focal and diffuse hyperfluorescence on FA [27].

In our study, well-defined leakage points identified in midphase FA images were treated without overlapping intact retinal areas in monospot mode. This laser therapy technique targets well-defined leakage points, especially those in the fovea, making SML treatment more precise and safer. In diffuse leakage areas, SML treatment in monospot mode is also more delicate compared to pattern mode, reducing the risk of thermal damage to the intact retinal pigment epithelium. Thus, this study showed that a personalized approach to SML therapy in patients with chronic CSC may involve not only customizing power titration but also precise individual selection of retinal areas for treatment.

Limitations of the present study include the lack of a control group, a relatively short follow-up (six months), and inclusion in the study of patients with different duration of the disease and various clinical manifestations. This study included patients with varying degrees of RPE involvement and decompensation. In addition, a limitation of this study can be considered, i.e., the lack of clear criteria for choosing a position on the retina suitable for titrating laser power. Therefore, a randomised trial within these limitations is appropriate to test the long-term safety and efficacy of 577 nm SML therapy with individual power settings in patients with chronic CSC.

In conclusion, SML therapy with a 577 nm laser targeted to the RPE and individual titration of laser power in the micropulse mode is a safe and effective treatment for patients with chronic CSC. SML therapy in patients with chronic CSC leads to complete or partial regression of SRF in 93% of cases within a 6-month follow-up period. Further studies are needed to test the long-term safety and efficacy of 577 nm SML therapy with individual power settings in chronic CSC.

## Figures and Tables

**Table 1:** Dynamics of functional and structural characteristics of patients with chronic CSC after SML therapy.

Indicators	Baseline *n* = 30	Month 1 *n* = 30	Month 3 *n* = 30	Month 6 *n* = 30
Mean ± SD decimal BCVA	0.35 ± 0.16	0.37 ± 0.1	0.4 ± 0.14	0.45 ± 0.15
*p* value		0.07	0.03	0.001
Mean ± SD CRT (*µ*m)	285 ± 76	264 ± 53	246 ± 47	236 ± 45
*p* value		0.01	0.001	0.003
Mean ± SD SRFH (*µ*m)	311 ± 85	185 ± 72	80 ± 45	45 ± 25
*p* value		0.03	0.001	0.001

BCVA, best-corrected visual acuity; CRT, central retinal thickness; SRFH, maximum subretinal fluid height.

## Data Availability

The data used to support the findings of this study are available from the corresponding author upon reasonable request.

## References

[1] Piccolino F. C., de la Longrais R. R., Ravera G. (2005). The foveal photoreceptor layer and visual acuity loss in central serous chorioretinopathy. *American Journal of Ophthalmology*.

[2] van Rijssen T. J., van Dijk E. H. C., Yzer S. (2019). Central serous chorioretinopathy: towards an evidence-based treatment guideline. *Progress in Retinal and Eye Research*.

[3] Grzybowski A., Sulaviková Z., Gawęcki M., Kozak I. (2024). Subthreshold laser treatment in retinal diseases: A mini review. *Graefe’s Archive for Clinical and Experimental Ophthalmology*.

[4] Clemente L., Cennamo G., Montorio D., Fossataro F., Passaro M. L., Costagliola C. (2022). OCT-A in chronic central serous chorioretinopathy treated with oral eplerenone and half-fluence photodynamic therapy: A comparative study. *European Journal of Ophthalmology*.

[5] Schmidt-Erfurth U., Laqua H., Schlötzer-Schrehard U., Viestenz A., Naumann G. O. (2002). Histopathological changes following photodynamic therapy in human eyes. *Archives of Ophthalmology*.

[6] Schlötzer-Schrehardt U., Viestenz A., Naumann G. O., Laqua H., Michels S., Schmidt-Erfurth U. (2002). Dose-related structural effects of photodynamic therapy on choroidal and retinal structures of human eyes. *Graefes Archive for Clinical and ExperimentalOphthalmology*.

[7] Chan W. M., Lam D. S., Lai T. Y., Tam B. S., Liu D. T., Chan C. K. (2003). Choroidal vascular remodelling in central serous chorioretinopathy after indocyanine green guided photodynamic therapy with verteporfin: A novel treatment at the primary disease level. *British Journal of Ophthalmology*.

[8] Kim S. W., Oh J., Oh I. K., Huh K. (2009). Retinal pigment epithelial tear after half fluence PDT for serous pigment epithelial detachment in central serous chorioretinopathy. *Ophthalmic Surgery, Lasers and Imaging Retina*.

[9] Nicholson B., Noble J., Forooghian F., Meyerle C. (2013). Central serous chorioretinopathy: Update on pathophysiology and treatment. *Survey of Ophthalmology*.

[10] Ratanasukon M., Thongthong K., Bhurayanontachai P., Jirarattanasopa P. (2013). Photoreceptor disruption in central serous chorioretinopathy treated by half-dose photodynamic therapy. *Clinical Ophthalmology*.

[11] Sirks M. J., van Dijk E. H. C., Rosenberg N. (2022). Clinical impact of the worldwide shortage of verteporfin (Visudyne®) on ophthalmic care. *Acta Ophthalmologica*.

[12] Li X., Long H., Hu Q. (2022). Efficacy of subthreshold micropulse laser for chronic central serous chorioretinopathy: A meta-analysis. *Photodiagnosis and Photodynamic Therapy*.

[13] Gawęcki M. (2019). Micropulse laser treatment of retinal diseases. *Journal of Clinical Medicine*.

[14] Scholz P., Ersoy L., Boon C. J., Fauser S. (2015). Subthreshold micropulse laser (577 nm) treatment in chronic central serous chorioretinopathy. *Ophthalmologica*.

[15] Luttrull J. K. (2016). Low-intensity/high-density subthreshold diode micropulse laser for central serous chorioretinopathy. *Retina*.

[16] Semenyuk V. (2017). Thermal interaction of multi-pulse laser beam with eye tissue during retinal photocoagulation: analytical approach. *International Journal of Heat and Mass Transfer*.

[17] Fedchenko S. A., Zadorozhnyy O. S., Molchaniuk N. I., Korol A. R. (2017). Ultrastructural changes in the rabbit chorioretinal complex following 577-nm laser photocoagulation. *Journal of Ophthalmology (Ukraine)*.

[18] Schwartz R., Habot-Wilner Z., Martinez M. R. (2017). Eplerenone for chronic central serous chorioretinopathy - a randomized controlled prospective study. *Acta Ophthalmologica*.

[19] Arsan A., Kanar H. S., Sonmez A. (2018). Visual outcomes and anatomic changes after sub-threshold micropulse yellow laser (577-nm) treatment for chronic central serous chorioretinopathy: Long-term follow-up. *Eye*.

[20] Maruko I., Koizumi H., Hasegawa T., Arakawa H., Iida T. (2017). Subthreshold 577 nm micropulse laser treatment for central serous chorioretinopathy. *PLoS One*.

[21] Chhablani J., Roh Y. J., Jobling A. I. (2018). Restorative retinal laser therapy: Present state and future directions. *Survey of Ophthalmology*.

[22] Gawęcki M., Jaszczuk-Maciejewska A., Jurska-Jaśko A., Grzybowski A. (2017). Functional and morphological outcome in patients with chronic central serous chorioretinopathy treated by subthreshold micropulse laser. *Graefes Archive for Clinical and Experimental Ophthalmology*.

[23] Lavinsky D., Palanker D. (2015). Nondamaging photothermal therapy for the retina: initial clinical experience with chronic central serous retinopathy. *Retina*.

[24] Lavinsky D., Sramek C., Wang J. (2014). Subvisible retinal laser therapy: Titration algorithm and tissue response. *Retina*.

[25] Yadav N. K., Jayadev C., Mohan A. (2015). Subthreshold micropulse yellow laser (577 nm) in chronic central serous chorioretinopathy: Safety profile and treatment outcome. *Eye*.

[26] Inagaki K., Ohkoshi K., Ohde S., Deshpande G. A., Ebihara N., Murakami A. (2015). Comparative efficacy of pure yellow (577-nm) and 810-nm subthreshold micropulse laser photocoagulation combined with yellow (561-577-nm) direct photocoagulation for diabetic macular edema. *Japanese Journal of Ophthalmology*.

[27] Chhablani J., Chhablani J., Ong J. (2022). Subthreshold laser therapy guidelines for retinal diseases. *Eye*.

